# Lipid metabolism reprogramming in tumor-associated macrophages and implications for therapy

**DOI:** 10.1186/s12944-023-01807-1

**Published:** 2023-03-31

**Authors:** Xuehan Qiao, Zhangmin Hu, Fen Xiong, Yufei Yang, Chen Peng, Deqiang Wang, Xiaoqin Li

**Affiliations:** 1 Department of Medical Oncology, The Affiliated Hospital of Jiangsu University, Zhenjiang, China; 2 Institute of Digestive Diseases, Jiangsu University, Zhenjiang, China

**Keywords:** Tumor-associated macrophages, Lipid metabolism, Immunotherapy, Chemotherapy resistance

## Abstract

The tumormicroenvironment (TME) plays a key role in tumor progression. Tumor-associated macrophages (TAMs), which are natural immune cells abundantin the TME, are mainly divided into the anti-tumor M1 subtype and pro-tumor M2 subtype. Due to the high plasticity of TAMs, the conversion of the M1 to M2 phenotype in hypoxic and hypoglycemic TME promotes cancer progression, which is closely related to lipid metabolism. Key factors of lipid metabolism in TAMs, including peroxisome proliferator-activated receptor and lipoxygenase, promote the formation of a tumor immunosuppressive microenvironment and facilitate immune escape. In addition, tumor cells promote lipid accumulation in TAMs, causing TAMs to polarize to the M2 phenotype. Moreover, other factors of lipid metabolism, such as abhydrolase domain containing 5 and fatty acid binding protein, have both promoting and inhibiting effects on tumor cells. Therefore, further research on lipid metabolism in tumors is still required. In addition, statins, as core drugs regulating cholesterol metabolism, can inhibit lipid rafts and adhesion of tumor cells, which can sensitize them to chemotherapeutic drugs. Clinical studies on simvastatin and lovastatin in a variety of tumors are underway. This article provides a comprehensive review of the role of lipid metabolism in TAMs in tumor progression, and provides new ideas for targeting lipid metabolism in tumor therapy.

## Introduction

**Fig. 1:**
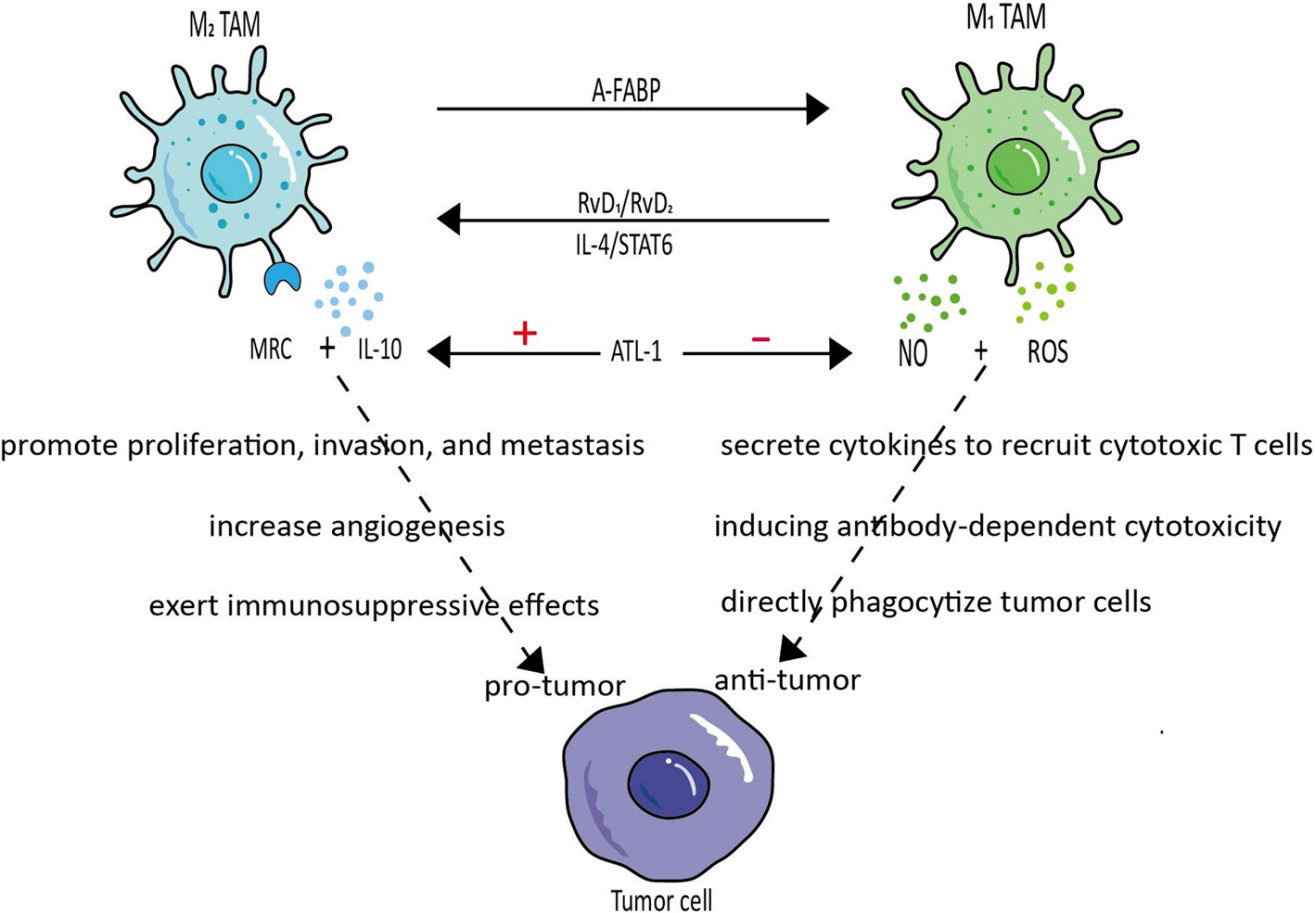
M1 TAMs and M2 TAMs are interconvertible. MRC and IL-10 are surface markers of M2 TAMs. NO and ROS are surface markers of M1 TAMs. They have almost opposite effects on tumors

The tumormicroenvironment (TME) is composed of immune cells (tumor-associated macrophages, natural killer cells, and lymphocytes), stromal cells and the extracellular matrix. They interact to provide an energy source for tumors and regulate tumor cell progression through multiple signaling pathways [1]. Tumor-associated macrophages (TAMs), one of the most common immune-infiltrating cells in the TME, play a key role in tumor development. TAMs are mainly divided into the classically activated pro-inflammatory M1 phenotype and alternatively activated anti-inflammatory M2 phenotype [2]. M1 TAMs are associated with type 1 immune responses driven by type 1 T helper cells (TH1) and innate lymphoid cells (ILC). The response can be activated by secreting cytokines, such as tumor necrosis factor-α (TNF-α), reactive oxygen species (ROS) recruitment of cytotoxic T cells [3], induction of antibody-dependent cytotoxicity [4] and direct phagocytosis of tumor cells for anti-tumor purposes (Fig.1) [5]. After secretion of anti-inflammatory cytokines by M1 TAMs at the site of injury, M2 TAMs can be recruited to the primary tumor site, where type 2 ILC and TH2 reaction products such as interleukin (IL)-4 and IL-13 are present. These products drive type 2 immune responses and activate M2 TAMs in the TME to promote tumor cell proliferation, invasion and metastasis, increase vascular and lymphatic vessel production and enhance tumor immunosuppression [6]. TAMs tend to have a M2 phenotype and exhibit pro-cancer activity in the TME. Since TAMs are highly plastic, they can be induced to polarize toward the M1 phenotype for anti-tumor purposes.

An important hallmark of cancer metabolism is the lipogenic phenotype [7]. The lipogenic phenotype means that the expression of lipogenic enzymes and key factors involved in the ab initio synthesis of fatty acids (FAs) is upregulated in malignant tumors, thus providing a growth advantage to tumor cells [8]. Lipid metabolism includes anabolism and catabolism (β-oxidation) and an imbalance between them can lead to lipid accumulation. Lipid accumulation has been observed in a variety of cancers, including breast, colorectal and ovarian cancers and is strongly associated with poor prognosis [9]. For example, the expression of fatty acid synthase (FASN) in triple-negative breast cancer (TNBC) is upregulated, causing lipid accumulation, which is usually positively correlated with cisplatin resistance. FASN can also function as an oncogenic protein to prevent cisplatin-induced apoptosis [10]. Lipid metabolism also provides energy to tumor cells. For example, fatty acid oxidation (FAO) provides reduced nicotinamide adenine dinucleotide (NADH), flavin adenine dinucleotide (FADH2), reduced nicotinamide adenine dinucleotide phosphate (NADPH) and adenosine triphosphate (ATP), which are essential sources of energy for tumor cells under hypoxic conditions [11]. The content of free fatty acids (FFA) in cancer cells is positively correlated with their aggressiveness. For example, in prostate cancer, increased FFA uptake by tumor cells activates the hypoxia-inducible factor (HIF)1/ matrix metalloproteinase 14 (MMP14) signaling pathway to enhance the invasive ability of tumor cells [12].

TAM metabolism includes glucose metabolism, lipid metabolism and amino acid metabolism. M1 TAMs preferentially use glucose to undergo glycolysis for energy supply, whereas M2 TAMs tend to rely on mitochondrial fatty acid oxidation to maintain energy balance [13, 14]. Owing to nutritional limitations in tumors, hypoxic and low-glycemic TME allow lipid metabolism to dominate tumor progression. Lipid metabolism in TAMs is closely associated with immunosuppression and resistance to chemotherapy. For example, in a mouse model of gastric cancer, lipid aggregation in TAMs upregulated the expression of phosphoinositide 3-kinase (PI3K-γ) and promoted TAMs polarization to the M2 phenotype. This resulted in decreased phagocytosis and upregulation of programmed death ligand 1 (PD-L1) expression in TAMs, attenuating the immune response of anti-tumor T cells, thus enhancing immunosuppression [15]. Resistance to chemotherapy is a serious challenge in current oncology treatments. In colorectal cancer, chemotherapy induces ROS activation of HIF1α, which drives the transcription of high-mobility group protein B1 (HMGB1) in tumor cells, thereby promoting macrophage infiltration in tumors. In addition, TAMs induced by colorectal cancer in mice produce high levels of growth differentiation factor 15 (GDF15), which enhances β-oxidation of fatty acids, thereby reducing the chemosensitivity of colon cancer cells to 5-FU [16].

In this paper, we provide detailed evidence that in hypoxic and hypoglycemic TME, TAMs tend to use lipid metabolic pathways to supply energy, thereby promoting or inhibiting tumor development. As lipid accumulation in TAMs allows M1 to M2 phenotypic differentiation, tumor promotion predominates. In addition, we present therapeutic approaches targeting the lipid metabolism of TAMs, including novel drugs and new applications of existing drugs, to enhance immunotherapeutic effects and improve chemotherapy resistance.

## Tumor regulation by lipid metabolism in TAMs

### Lipid metabolism involving fatty acids and their derivatives in TAMs

#### Lipid metabolism involving peroxisome proliferator-activated receptors in TAMs

The peroxisome proliferator-activated receptor (PPAR) is involved in almost the entire process of fatty acid metabolism [17]. Three PPAR isoforms have been identified: PPARα, PPARγ and PPARβ/δ. PPARα and PPARδ mainly regulate oxidative phosphorylation, substrate transport and energy balance, while PPARγ plays an essential role in lipid synthesis [18]. PPAR can regulate the polarization of TAMs to the M2 phenotype through multiple cellular pathways, which in turn promotes tumor cell proliferation, invasion angiogenesis and immunosuppression.

**Fig. 2:**
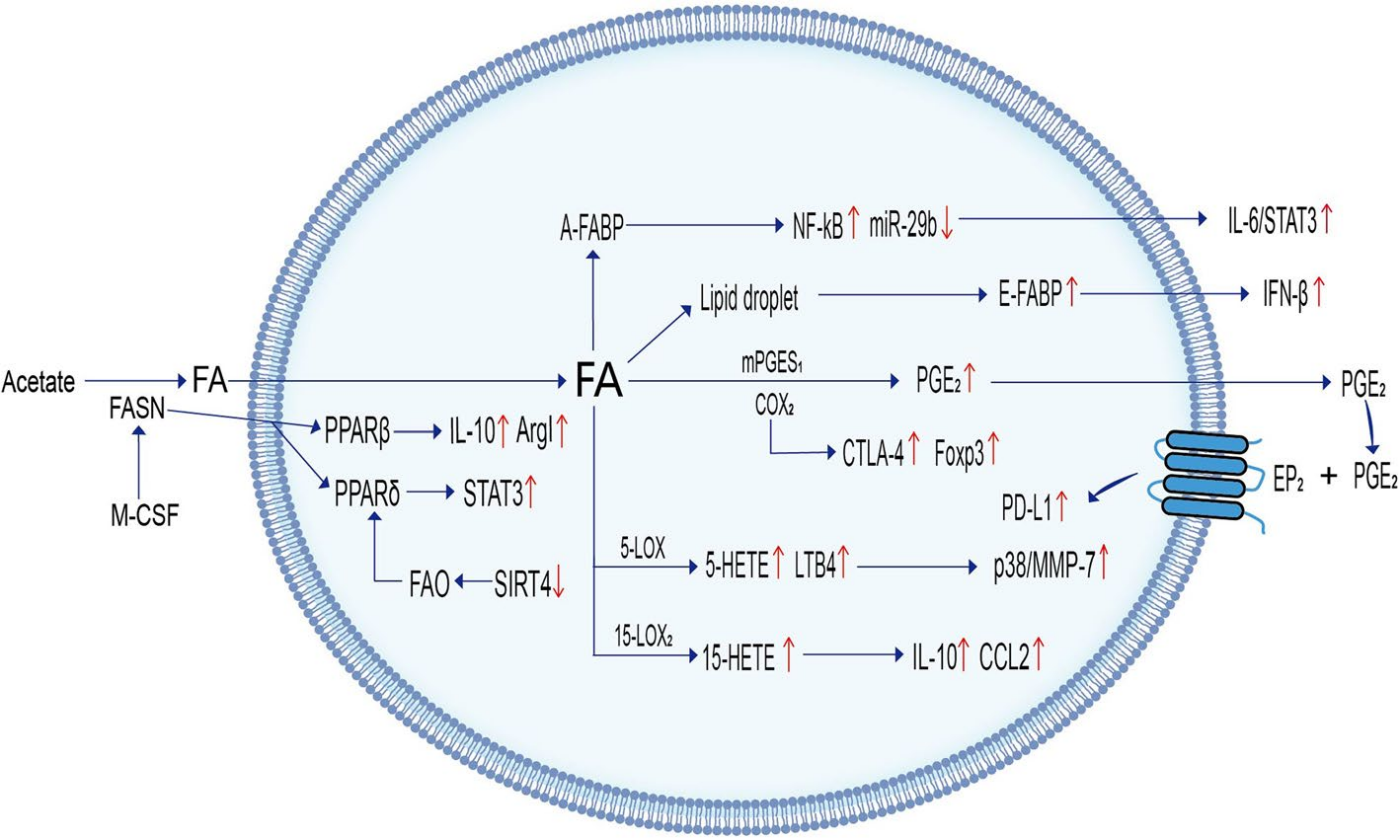
Lipid metabolism of tumor-associated macrophages. Up arrows indicate upward adjustment. Down arrows indicate downward adjustment

PPARβ/δ involved in lipid metabolism can promote the activation of TAMs toward the M2 phenotype and enhance the invasiveness and angiogenesis of tumor cells. For example, macrophages isolated from human serous ovarian carcinoma ascites containing high concentrations of polyunsaturated fatty acids such as linoleic acid can act as fatty acid ligands to effectively agonize PPARβ/δ, thus contributing to the pro-tumor polarization of TAMs and promoting tumor cell invasion and dissemination [19]. In a mouse model of Lewis lung cancer, tumor cell secretion of macrophage colony-stimulating factor-induced FASN upregulation in M2 TAMs activated tumor myeloid cells to express PPARβ/δ, leading to increased release of immunosuppressive cytokines such as IL-10 and arginase I (Arg I), ultimately promoting tumor cell invasion and angiogenesis [20]. SIRT4, a member of the sirtuin family (SIRT1-7), can influence cell proliferation and metabolic regulation, exerting the role of a tumor growth suppressor [21]. Downregulation of SIRT4 in TAMs derived from human hepatocellular carcinoma activates the FAO-PPARδ-signal transducer and activator of transcription (STAT)3 signaling pathway, which regulates the polarization of TAMs toward the M2 phenotype by increasing FA oxidation (Fig.2) [22].

Niu et al. [23] found that the proliferation and immunosuppression of tumor cells are closely linked to PPARγ-mediated lipid metabolism. Caspase-1/tPPARγ/medium-chain acyl-CoA dehydrogenase (MCAD) signaling axis plays an important role in the differentiation of TAMs isolated from mouse breast cancer tissues. Caspase-1 specifically cleaves PPARγ in breast cancer cells. Truncated PPARγ undergoes cytoplasmic-mitochondrial translocation, which inhibits MCAD and FAO and promotes lipid accumulation, leading to reprogramming of TAMs to the M2 phenotype and proliferation of tumor cells [23]. The use of PPARγ inhibitors attenuates the pro-inflammatory effect of M1 TAMs and inhibits the secretion of pro-tumor cytokines by M2 macrophages to achieve the goal of inhibiting tumor progression [24, 25]. In addition, the caspase inhibitor NCX-4016 is widely used in the treatment of mouse colon cancer, which can inhibit caspase-1 activity and target TAMs to weaken pro-tumorigenic activity, thereby inducing apoptosis and inhibiting the growth of tumor cells [23, 26]. However, in human and mouse hepatocellular carcinoma tissues, downregulation of receptor-interacting protein kinase 3 (RIPK3), a central factor of necroptosis in macrophages, significantly inhibits caspase-1-mediated cleavage of PPAR, promotes fatty acid oxidation, polarizes TAMs toward the M2 phenotype and enhances M2 TAM-mediated immunosuppression. It has been shown that hypomethylation of RIPK3 by decitabine inhibits FAO and reverses the pro-tumor polarization of TAMs [27].

#### Lipid metabolism involved in fatty acid binding proteins in TAMs

Fatty acid-binding protein (FABP), an intracellular lipid chaperone, coordinates lipid distribution and is a key mediator in regulating intracellular fatty acid metabolism and inflammatory pathways [28]. TAM expression of adipocyte/macrophage fatty acid binding protein (A-FABP) is increased in human and mouse breast cancer tissues. The upregulation of A-FABP can enhance the signal transduction of IL-6/ STAT3 by upregulating nuclear factor-kappa B (NF-κB) and downregulating the expression of miR-29b, thus promoting the growth and metastasis of breast cancer cells [29]. Interestingly, in human and mouse breast cancer models, epidermal fatty acid-binding protein (E-FABP) has a tumor-killing effect. In the early stages of tumor development, macrophage-tumor interactions promote E-FABP-mediated lipid droplet formation as well as macrophage energy storage, enhance the effect of macrophage immunosurveillance and recruit natural killer cells to the tumor mesenchyme to kill tumor cells [30]. A compound named EI-05, which acts as an E-FABP activator, has been found to increase lipid droplets and interferon (IFN) -β production in TAMs isolated from mouse breast cancer, thus boosting the anti-tumor activity of TAMs [31]. This shows that different phenotypes of FABP can both promote tumor growth and metastasis and enhance tumor-killing effects. Therefore, it is necessary to have precise tumor treatment targets to have good therapeutic effects on tumors.

#### Lipid metabolism involving CD36 in TAMs

CD36 is highly expressed in human tumor tissues and is a key receptor for lipid uptake by macrophages. CD36 also acts as a prognostic marker for a variety of cancers, including ovarian, glioma and prostate cancers. TAMs express high levels of the scavenger receptor CD36 and use FAO rather than glycolysis to provide energy. High levels of FAO activate STAT6 and the transcription of genes that regulate the production and function of TAMs. Thus, CD36 expression is positively correlated with TAM production and FAO [9]. S100A4, a well-established premetastatic oncoprotein, is preferentially expressed by macrophages in the TME. The S100A4/PPARγ pathway enhances CD36-mediated FA uptake and thus the pro-tumor activity of TAMs [32]. Therefore, S100A4 may serve as a therapeutic target in tumors.

#### Lipid metabolism involving scavenger receptor A in TAMs

Scavenger receptor A (SR-A), also known as differentiation cluster 204 (CD204), is an essential marker of M2 TAMs. It has shown that tumor infiltrating CD204 M2 macrophages are closely related to the poor prognosis of various tumors [33]. SR-A plays an important role in lipid metabolism. The modified low density lipoprotein (LDL) has cytotoxicity which can induce inflammation and accumulation of lipid intima. SR-A absorbs the modified LDL through endocytosis [34]. Previous studies have shown that the content of cholesterol in blood and the level of modified LDL in mice with simultaneous knockout of SR-A and ApoE are lower than those with simple knockout of ApoE. It shows that SR-A plays a key role in the elimination of lipoproteins [35]. However, another study shows that SR-A deficiency can lead to more serious lesions and aggravate the formation of atherosclerosis [36]. Therefore, the role of SR-A in lipid metabolism requires further research.

### Lipid metabolism involving phospholipids in TAMs

Phospholipids are divided into two major groups, glycerophospholipids and sphingolipids. They are involved in the composition of cell membranes and various cellular signaling pathways. Arachidonic acid (AA) is a membrane phospholipid produced and released into the cytosol by phospholipase A2. The enzymes involved in AA metabolism include cytochrome P450 (CYP450), cyclooxygenase (COX) and lipoxygenase(LOX). AA produces hydroxyeicosatraenoic acids (HETEs), prostaglandins (PG) and leukotrienes (LTs), respectively, in response to these enzymes [37]. TAM phospholipid metabolism mainly regulates the immune escape and proliferation of tumor cells.

#### Lipid metabolism involving COX in TAMs

PD-L1 mediates the immune escape of tumor cells by suppressing activated programmed cell death protein 1 (PD-1)-positive T lymphocytes. It has been demonstrated that TAMs isolated from bladder cancer mice highly expressed PD-L1 and induced apoptosis of CD8 + T cells. Moreover, TAMs highly express COX2 (PGE2 forming enzyme) and microsomal PGE2 synthase 1 (mPGES1), which promote TAMs and tumor cells to secrete large amounts of immunosuppressive lipid PGE2. PGE2 can further upregulate PD-L1 expression. Thus, targeting mPGES1, PGE2 and COX2 can downregulate PD-L1 expression in TAMs and limit their immunosuppressive effect [38].

TAMs derived from mouse colorectal tumor secrete extracellular vesicles (EVs) that inhibit tumor progression. Enzymes associated with the AA pathway, such as COX1 and thromboxane synthase 1, have been identified in TAM-EVs. TAM-EVs shift the catabolism of AA from a COX2-dependent pathway to a COX1-dependent pathway, thereby inhibiting the tumor-promoting effects of prostaglandins (PGF2a and PGE2) [39]. This indicates that COX-related lipid metabolism in TAMs plays a bidirectional role in promoting tumor immunosuppression and inhibiting tumor progression.

#### Lipid metabolism involving LOX in TAMs

Lipid metabolism involving LOX in TAMs can promote the secretion of immunosuppressive factors by TAMs to mediate the immune escape of tumors. It has been demonstrated that TAMs isolated from kidney cancer patients with high expression of 15-lipoxygenase-2 (15-LOX2) stimulate the inflammatory cytokine CCL to recruit CCR2-expressing mononuclear myeloid cells. Mononuclear myeloid cells secrete large amounts of bioactive lipid products 15(S)-HETEs, which in turn promote the secretion of IL-10 by TAMs and enhance the immunosuppression and immune escape of tumor cells [40]. In addition, TAMs infiltrated by human renal cancer can upregulate COX-2 and increase the expression of cytotoxic T-lymphocyte-associated protein-4 (CTLA-4) and forkhead box protein 3 (Foxp3) [40, 41]. CTLA-4, as the second receptor of the B7 family of co-stimulatory molecules, can inhibit the activation of T cells and reduce the sensitivity of cancer cells to the immune killing effect of T cells, while the Foxp3 transcription factor is a key factor in dominant immune tolerance [42, 43]. Both can induce immune tolerance. Therefore, the production of immunosuppressive factors IL-10 and CCL2 by TAMs can be downregulated by inhibiting the activity of LOX in the TME, which also provides a new direction to enhance the effect of immunotherapy in kidney cancer patients.

Lipid metabolism involving phospholipids plays a regulatory role in the proliferation of tumor cells. Lipoxin (LX), a lipid mediator of LOX, can inhibit tumor cell growth. It has been shown that when human macrophages are co-cultured with human melanoma cells, ATL-1, an analogue of 15-epi-lipoxins (15-LX) A4, can selectively reduce the surface markers of M2 macrophages, including mannose receptors and IL-10 in TAMs, to enhance their tumoricidal activity. At the same time, ATL-1 can also induce apoptosis of human melanoma cells by triggering the production of nitric oxide (NO) and ROS. It was also confirmed in the mouse melanoma model that ATL-1 could inhibit tumor growth and the M2 TAMs markers isolated from mouse melanoma were significantly reduced [44]. Metabolites derived from ω-3 long-chain polyunsaturated fatty acids have anti-inflammatory and pro-tumor effects because of their ability to polarize M1 TAMs to the M2 type [45]. For example, docosahexaenoic acid is successively converted to docosahexaenoids, including catabolites D1 (resolvin D1, RvD1) and RvD2, in the presence of 15-LOX and 5-LOX [46]. In the mouse prostate cancer model, RvD1 and RvD2 promote the IL-4-STAT6 pathway-mediated polarization of TAMs toward the M2 phenotype and promote tumor cell proliferation. Therefore, targeting the RvDs-induced polarization of TAMs may be a therapeutic approach in prostate cancer [47]. The lipid metabolism involved in LOX in TAMs not only promotes the formation of an immunosuppressive microenvironment but also promotes the proliferation of tumor cells.

Cancer cells are characterized by a high proliferation rate and angiogenesis. Thus, a hypoxic TME is formed in tumors. In a hypoxic environment, HIF promotes FA uptake, synthesis and storage, contributing to fat accumulation. High expression of HIF is often positively correlated with poor prognosis in cancer [48]. The metabolites of 5-LOX, 5-HETE and LTB4 are increased in the hypoxic TME, enhancing the infiltration of TAMs in human ovarian cancer tissue via the p38/ MMP-7 pathway [49]. Zileuton, a specific and selective inhibitor of 5-LOX, reduces the expression of MMP-7, thus reducing the migration and infiltration of TAMs [49, 50].

### Lipid metabolism involving triglycerides in TAMs

Triglycerides are involved in both anabolic and catabolic metabolism. The anabolism is dependent on diacylglycerol O-acyltransferases (DGAT) and monoacylglycerol O-acyltransferases (MGAT) and the catabolism-related enzymes include hormone-sensitive lipase (HSL), abhydrolase domain containing 5 (ABHD5), adipose triglyceride lipase (ATGL) and monoglyceride lipase (MGLL) [51]. Triglyceride-related lipid metabolism in TAMs can inhibit tumor cell apoptosis and enhance immunosuppression.

#### Lipid metabolism involving ABHD5 in TAMs

**Fig. 3:**
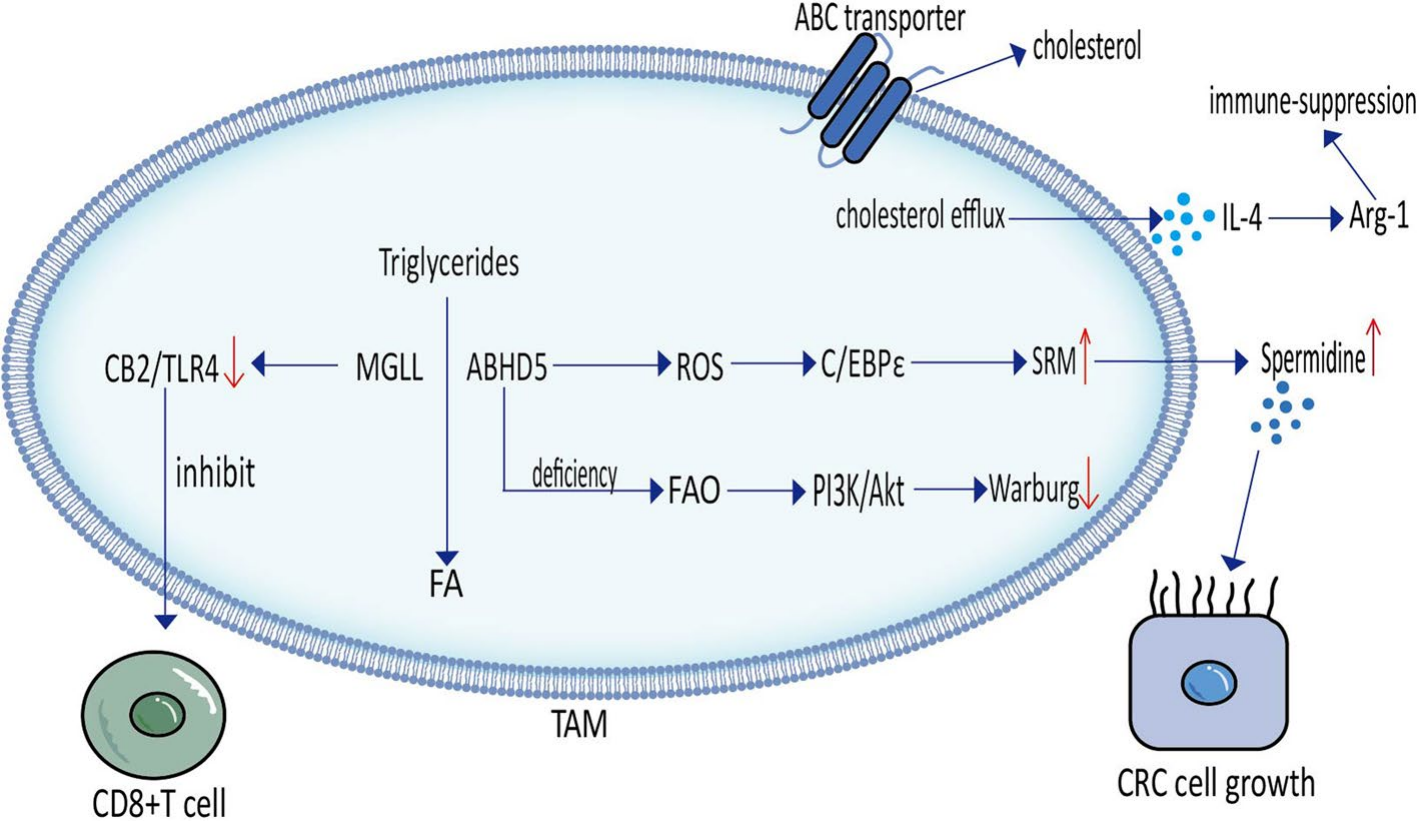
Triglyceride and Cholesterol metabolism of tumor-associated macrophages. Up arrows indicate upward adjustment. Down arrow indicate downward adjustment

ABHD5, a key enzyme in triglyceride catabolism, inhibits autophagy and apoptosis in tumor cells [51]. Ectopic expression of ABHD5 in TAMs from human and mouse colon cancer tissues activates spermine synthase (SRM) transcription, which in turn inhibits spermidine production. Spermine, however, plays a role in promoting the apoptosis of tumor cells. Therefore, targeting the ABHD5/SRM/spermine axis of TAMs could be a potential therapeutic strategy for colon cancer [52]. However, in colon cancer, OU et al. found that ABHD5 deficiency inhibited mitochondrial FAO in TAMs isolated from human and mouse tumor tissues, activated the PI3K/protein kinase B (PKB/Akt) pathway and promoted aerobic glycolysis (Warburg effect), which in turn inhibited AMP-activated protein kinase phosphorylation and p53 activity. Eventually, the autophagy of tumor cells was inhibited (Fig.3) [53]. Therefore, the role of ABHD5 in tumors requires further study.

#### Lipid metabolism involving MGLL in TAMs

MGLL is a key enzyme in triglyceride catabolism that hydrolyzes triglycerides into FFAs. In the mouse model of colon cancer and breast cancer, MGLL deficiency leads to lipid accumulation in TAMs and promotes endocannabinoid receptor-2 (CB2)/ Toll-like receptor (TLR)4 activation of M2 TAMs, thereby enhancing immunosuppression. Therefore, CB2 antagonists can be used to maintain CD8 + T cell activity and delay tumor progression [54]. A reduction-responsive RNAi nanoplatform has been developed to silence both MGLL and CB2 to inhibit the production of FFAs, which promotes the classical activation of TAMs to secrete tumor-killing cytokines, such as TNF-α and IL-12, to exert an inhibitory effect on pancreatic cancer [55].

### Lipid metabolism involving cholesterol in TAMs

Cholesterol is an important component of biological membranes and is produced as an isoprene precursor via the mevalonate pathway. It regulates membrane fluidity and participates in various signaling pathways as a solubilizing agent for other lipids. Cholesterol in the TME can suppress adaptive and innate immune responses, causing the depletion of CD8 + T cells. It plays an important role in tumor immunosuppression and cancer cell proliferation. Cholesterol metabolism reprogramming can regulate the activity and recruitment process of TAMs which can promote the polarization of TAMs to M2 phenotype and play an important role in regulating tumor progression. At present, the metabolism of cholesterol in TAMs mainly focuses on the change of cholesterol efflux pathway. Therefore, the efflux of targeted cholesterol may become a potential method to treat tumors and the relationship between cholesterol and TAMs needs further study [56, 57].

#### Lipid metabolism involving the ATP-binding cassette in TAMs

The adenosine triphosphate-binding cassette (ABC) transporter removes excess intracellular cholesterol and maintains dynamic cholesterol homeostasis. Cholesterol enrichment in tumor tissues has been detected in a variety of tumors, whereas TAMs isolated from tumor tissues exhibit cholesterol deficiency [58, 59]. Lipid metabolism involved in ABC in TAMs promotes tumor immunosuppression. However, the lack of ABC transporters in TAMs and the use of ABC inhibitors can suppress tumors by directly killing tumor cells and inhibiting tumor angiogenesis.

In a mouse model of metastatic ovarian cancer, ABC transporters mediated membrane cholesterol efflux from TAMs, polarizing TAMs into tumor-promoting M2 phenotype, promoting IL-4-associated immunosuppression and invasive metastasis and inhibiting IFN-γ-induced anti-tumor effects [60]. Similarly, in bladder cancer and melanoma mouse models, ABCG1, a member of the ABC transporter family, regulated cholesterol efflux from TAMs. ABCG1-/- TAMs exhibit intracellular free cholesterol (FC) accumulation and polarization toward the M1 phenotype, activating the IκB kinase/NF-κB pathway, which has a direct killing effect on tumor cells, ultimately inhibiting tumor growth [58]. The ABC inhibitor ATR101 has been suggested to inhibit cholesterol efflux from TAMs, which in turn suppresses TAM-induced angiogenesis and chemotaxis [59]. ATR101 also inhibits acyl-coenzyme A: cholesterol O-acyltransferase 1 (ACAT1), thereby inhibiting the esterification of FC and attenuating FC accumulation to promote the apoptosis of human adrenocortical carcinoma cells [61]. Therefore, inhibiting the efflux of TAMs cholesterol and mediating the activation of TAMs to M1 phenotype may become one of the treatment directions of future tumors.

TAMs isolated from liver metastases of human colon cancer also upregulate liver X receptor (LXR) genes and downstream genes of LXR involved in cholesterol transport, such as the ABC family, enzymes involved in lipid metabolism and extracellular lipid receptors and downregulate genes related to lipid uptake and synthesis, which may be associated with the formation of an immunosuppressive microenvironment [62].

#### Lipid metabolism involving 27-hydroxycholesterol in TAMs

Cholesterol can promote the activation of TAMs. 27-hydroxycholesterol (HC) is the main metabolite of cholesterol under the catalysis of cytochrome P450 oxidase (CYP27A1). CYP27A1 expression in M2 TAMs is significantly higher than M1 phenotype and can activate M2 TAMs and promote the progress of breast cancer [63]. In addition, when co cultured with breast cancer, human monocytic leukemia(THP-1) cells are more likely to differentiate into M2 TAMs. 27-HC synthase CYP27A1 is highly expressed in M2 TAMs derived from breast cancer in mice, whereas the 27-HC catabolic enzyme CYP27B1 is expressed at low levels in tumor cells, leading to the accumulation of 27-HC in tumor tissues. The aggregation of 27-HC stimulates the proliferation of estrogen receptor-positive breast cancer cells, promotes the expression of various chemokines such as CCL2 and CCL3 by TAMs and recruits monocytes to the primary tumor site, which in turn promotes the development of breast cancer [64]. Therefore, 27-HC can not only promote the proliferation of cancer cells, but also change the phenotype and number of macrophages to accelerate tumor progression. It is an important factor between tumor cells and TAMs.

#### Lipid metabolism involving apolipoprotein E in TAMs

Apolipoprotein E (Apo E) is secreted at high levels by hepatocytes and macrophages and mediates cholesterol metabolism. Moreover, Apo E has the opposite effects of mediating immunosuppression and enhancing cytotoxic T-cell responses due to different TMEs in different tumors [65]. In human and mouse pancreatic ductal adenocarcinoma (PDAC), TAMs expressing high levels of Apo E can bind to low-density lipoprotein (LDL) receptors on the surface of tumor cells, which activates the NF-κB pathway and promotes the secretion of chemokines CXCL1 and CXCL5 (chemoattractants for immature myeloid cells) by cancer cells to inhibit CD8 + T-cell immune infiltration, ultimately promoting an immunosuppressive TME [66]. Therefore, targeting Apo E or LDL receptors on the tumor surface may provide new ideas for tumor immunotherapy.

#### Lipid metabolism involving high-density lipoprotein in TAMs

As plasma lipoproteins, high-density lipoprotein (HDL) acts as a carrier in the reverse transport of cholesterol which can participate in the structural destruction of lipid rafts of mouse and human primary macrophages and the depletion of membrane cholesterol. It activates the PKC/NF-κB/STAT1 axis, thus enhancing the signal transduction mediated by TLR and ultimately promotes expression of macrophage inflammatory factors (such as TNF- α) [67]. ApoA-I, as a component of HDL, is a key medium for plasma cholesterol transport and maintains cell cholesterol homeostasis. Some studies have shown that infusion of ApoA-I into the melanoma model of mice can make TAMs change from M2 to M1 phenotype, promote the increase of IFN-γ and the accumulation of CD8 + T cells to the growth and metastasis of tumor [68]. In addition, M2 TAMs express SR-A, as an ApoA-I analog, a small peptide inhibitor (D-amino acid peptide), can be used for targeted treatment in vivo to block SRA-mediated adhesion of TAMs and prevent tumor progression and distant metastasis [69].

### Other relevant lipid metabolism in TAMs

Recently, it was shown that immune cells are also involved in the lipid metabolism process in TAMs. Regulatory T (Treg) cells can inhibit the secretion of IFN-γ by CD8 + T cells, which in turn promotes cholesterol regulatory element-binding protein 1 (SREBP1)-mediated fatty acid synthesis in M2 TAMs derived from mouse melanoma and colon cancer. In addition, Treg cell inhibition of IFN-γ limits the spare respiratory capacity of M1 TAMs, thus enhancing the metabolic effects of M1 TAMs. Therefore, the therapeutic effect of immune checkpoint blockade can be enhanced by inhibiting SREBP1, suggesting that targeting Treg cells may improve the immunotherapeutic effect in cancer [70].

## Implications for guidance of tumor treatment

In recent years, significant advances have been made in research on lipid metabolism. Targeting lipid metabolism in TAMs and inducing the conversion of TAMs to the M1 phenotype may enhance the effect of tumor immunotherapy and reduce chemotherapy resistance (Table1) [71].

**Table 1:** Drugstargeting lipid metabolism in TAMs

Drug	Target	Metabolic pathway/consequence	Clinical trials	Phase	Disease model
**Enhance immunotherapy effect**					
Fatostatin [70]	SREBP1	Inhibit de novo synthesis of fatty acids in TAMs			Melanoma
Etomoxir [72]	CPT-1A	Inhibit FAO-dependent ROS and NOD-like receptor thermoprotein			Liver Cancer
RQ treatment [73]	Cellular autophagy	Increase ratio of M1/M2 TAMs and CD8/CD4 + T cells			Glioblastoma
IP549 [15]	PI3K-γ	Reduce lipids in TAMs			Gastric Cancer
Decitabine [27]	Inhibit FAO	Inhibit pro-tumor polarization of TAMs			Liver Cancer
**Improve chemotherapy resistance**					
SB431542 [16]	SMAD signaling pathway	Inhibit SMAD-GDF15-FAO			Colorectal Cancer
GB111-NH2 [74]	Histone	Inhibit histones B, L and S			
C75 [10]	FASN	Enhance cisplatin-induced apoptosis in tumor cells			TNBC
Simvastatin [75]	HMG-CoA reductase	Inhibit integrin/FAK/ERK signaling pathway and EMT	NCT00452244 NCT00452634 NCT01441349 NCT04985201 NCT04698941 NCT01156545 NCT02197234	Phase 2 Phase 2 Phase 2 Phase 2 Phase 2 Phase 2 Phase 1	Lung Cancer
Lovastatin [76]	HMG-CoA reductase	Downregulate IL-10 and upregulate IFN-γ	NCT00285857 NCT00902668 NCT00584012	Phase 2 Phase 2 Phase 2	Breast Cancer
**Other drugs**					
Zileuton [49, 50]	5-LOX	Reduce MMP-7 and inhibit TAMs migration and infiltration	NCT00056004 NCT00070486	Phase 2 Phase 2	Ovarian Cancer Lung Cancer
NCX-4016 [23]	Caspase-1	Induce apoptosis and inhibit the growth of tumor cells	NCT00331786	Phase 1	Colon Cancer
ATR101 [59]	ABC	Inhibit cholesterol efflux from TAMs			Lung Cancer

### Enhanced immunotherapy effect

Targeting the synthesis and catabolism of fatty acids in TAMs can enhance immunotherapeutic effects. The immune checkpoint PD-1 in tumors alternatively activates TAMs into the M2 phenotype and the phagocytosis of macrophages is reduced [77]. In mouse models of melanoma and colon cancer, SREBP1 mediates ab initio synthesis of fatty acids in M2 TAMs. Fatostatin, an SREBP1 inhibitor, selectively inhibits the activation of M2 TAMs and activates CD8 + T cells, triggering a positive feedback loop favoring immune stimulation of the TME. The combination of fatostatin with anti-PD-1 drugs can increase the blocking efficiency of immune checkpoint inhibitors for tumor growth inhibition [70]. Carnitine palmitoyl transferase (CPT), a key rate-limiting enzyme of FAO, can bind fatty acids and carnitine into the mitochondria, thereby regulating FAO and promoting the metabolic response in cancer [9]. Etomoxir (a CPT-1A inhibitor) inhibits FAO-dependent ROS and NOD-like receptor thermoprotein structural domain-associated protein 3 in M2 TAMs, which in turn inhibits IL-1β secretion and limits TAM-mediated tumor cell migration [72]. In mouse models of lung and colorectal cancer, the use of FAO inhibitors enhanced the anti-tumor effects of pericyte therapy by suppressing tumor-infiltrating myeloid-derived suppressor cell (MDSC)-cell-mediated immunosuppression [78].

The combination of the autophagy inducer sirolimus (rapamycin, R) and the autophagy inhibitor hydroxychloroquine (Q) can inhibit the activation of M2 TAMs by inhibiting their lipid utilization. The combination of RQ treatment with anti-PD-1 also enhanced immunotherapeutic efficacy by increasing the expression of CD47 and Sirpα on the surface of tumor cells and macrophages, thereby enhancing the phagocytic capacity of macrophages [73]. In a mouse model of soft tissue sarcoma, the retinoblastoma (RB) tumor suppressor plays an important role in the TME and RB inactivation promotes tumor growth and neoangiogenesis. This effect is mediated through enhanced FAO activity, mitochondrial superoxide dismutation production and activation of the CCL2-CCR2 axis, which promotes the recruitment of immunosuppressive cells. Thus, inhibitors of the CCL2-CCR2 axis can be used to treat RB-deficient tumors and anti-CCL2 treatment increases the therapeutic effect of immune checkpoint blockade (ICB) [79]. In a mouse model of gastric cancer, treatment with the PI3K-γ inhibitor IP549 reduced the lipid levels in TAMs. The phagocytic capacity of macrophages and cytotoxic T cells that exert tumor-killing effects is enhanced [15]. It has been shown that targeting leukocyte immunoglobulin-like receptor B2 (LILRB2) can downregulate multiple genetic targets of M2 TAMs and change cholesterol metabolism in human monocytes, thereby enhancing immune effects [80].

### Improved chemotherapy resistance

Resistance to chemotherapy is a current challenge in cancer treatment. Several studies have targeted the lipid metabolism of TAMs to improve chemotherapy resistance in tumors. In the mouse model of colorectal cancer, 5-FU resistance is mainly due to the production of GDF15 by TAMs to enhance FAO, which reduces the chemosensitivity of tumors. Activation of GDF15 requires the SMAD signaling pathway. Therefore, chemosensitization can be achieved by either using SB431542 to inhibit the SMAD signaling pathway or etomoxir to inhibit FAO [16]. Recently, a new tissue protease inhibitor GB111-NH2 was found to pharmacologically inhibit histones B, L and S, resulting in increased expression of key regulators of fatty acids, such as FASN and acid ceramidase, to promote the polarization of M2 TAMs to M1 TAMs and reduce chemoresistance [74]. In contrast, the use of C75, an inhibitor of FASN, enhances cisplatin-induced apoptosis and improves cisplatin resistance in TNBC [10].

Cholesterol metabolism plays an important role in regulating the polarization of TAMs and epithelial-mesenchymal transition (EMT). Cardiovascular drugs that regulate cholesterol metabolism can be used for the chemosensitization of tumors and several clinical studies have demonstrated this effect. Jin [75] recently found that simvastatin, as a targeted hydroxymethylglutaryl coenzyme A (HMG-CoA) reductase inhibitor, in combination with paclitaxel, downregulated lipid rafts in tumor cells, inhibited adhesion and thus suppressed the integrin/FAK/ERK signaling pathway and EMT. The mechanism re-sensitized drug-resistant cancer cells to paclitaxel and inhibited polarization of M2 TAMs derived from mouse bone marrow and cholesterol homeostasis, reversing M2 TAMs to M1 TAMs. Statins also have immunomodulatory effects. In the mouse model of breast cancer, lovastatin downregulates IL-10 and upregulates IFN-γ expression in CD45 + cells, suggesting that M1 phenotype TAMs tend to exert anti-tumor effects. In addition, lovastatin increases the infiltration of effector T cells in tumors to attenuate immunosuppression and inhibit tumor angiogenesis induced by M2 TAMs [76]. Although statins have anti-tumor effects, the required dose is 100–500 times the anti-cholesterol dose, whereas simvastatin long-circulating liposome-encapsulated simvastatin (LCL-SIM) can specifically aggregate in tumor tissue and has tropism for TAMs. LCL-SIM led to a dramatic improvement in the anti-tumor efficacy of statins by inhibiting TAM-mediated oxidative stress and ultimately inhibiting mouse melanoma progression and invasion [81]. All above explained mechanisms indicate that cholesterol metabolism can be used as an effective target for tumor therapy to improve chemoresistance and immunotherapy efficacy in patients and the new use of old drugs can also reduce the cost of tumor treatment.

## Discussion

In recent years, lipid metabolism has become a hot research topic. Owing to the hyperproliferative nature of tumors, the resulting nutritional dysregulation and hypoxic TME allow lipid metabolism to dominate in tumors. TAMs, as immune-infiltrating cells in the TME, can interact with cancer cells. Lipid metabolites in TAMs can promote and inhibit tumor cell development. The promoting effects include enhancing immunosuppression of tumor cells, promoting proliferation, invasion and metastasis and inhibiting autophagy and apoptosis of tumor cells, while the inhibitory effect works mainly by enhancing immune surveillance. Moreover, tumor cells can also induce the upregulation of key genes of lipid metabolism in TAMs, promoting TAM lipid accumulation and inducing TAM polarization toward the M2 phenotype to further promote tumor progression. Thus, tumor development is closely related to interactions between the lipid metabolites of TAMs.

Owing to the essential role of lipid metabolism of TAMs in tumors, several studies have been conducted to exert oncogenic effects by targeting lipid metabolism in TAMs or inducing conversion of TAMs to anti-tumor phenotypes. However, the study of lipid metabolism in TAMs is not systematic, lipid metabolism initiation mechanism in TAMs is not yet understood and the mechanisms by which lipid metabolism may play opposite roles in the same carcinoma are unclear. Recent studies have shown that accumulation of lipids in other immune cells such as MDSC [82] and dendritic cells [83] has also been found to cause changes in their metabolism and function. Therefore, targeting immune cell metabolism provides new ideas for tumor therapy.

## Data Availability

Not applicable.

## Abbreviations

AA: Arachidonic acid
ABC: Adenosine triphosphate binding cassette
ABHD5: Abhydrolase domain containing 5
A-FABP: Adipocyte/macrophage fatty acid binding protein
Akt(PKB): Protein kinase B
Apo: Apolipoprotein
ATP: Adenosine triphosphate
CB2: Endocannabinoid receptor-2
COX: Cyclooxygenase
CTLA-4: Cytotoxic T lymphocyte-associated protein-4
CYP450: Cytochrome P450
E-FABP: Epidermal fatty acid binding protein
EMT: Epithelial-mesenchymal transition
FABP: Fatty acid binding protein
FAO: Fatty acid oxidation
FASN: Fatty acid synthase
FC: Free cholesterol
FFA: Free fatty acids
Foxp3: Forkhead box protein 3
GDF15: Growth differentiation factor 15
HETEs: Hydroxyeicosatraenoic acids
HDL: High density lipoprotein
ICB: Immune checkpoint blockade
IFN: Interferon
IL: Interleukin
LDL: Low density lipoprotein
LILRB2: Leukocyte immunoglobulin-like receptor B2
LOX: Lipoxygenase
LTs: Leukotrienes
LX: Lipoxin
LXR: Liver X receptor
MMP: Matrix metalloproteinase
MCAD: Medium-chain acyl-CoA dehydrogenase
MDSC: Myeloid-derived suppressor cell
MGLL: Monoglyceride lipase
mPGES1: Forming enzymes microsomal PGE2 synthase 1
NADPH: Reduced nicotinamide adenine dinucleotide phosphate
NF-κB: Nuclear factor-kappa B
NO: Nitric oxide
PD-1: Programmed cell death protein 1
PD-L1: Programmed death ligand 1
PG: Prostaglandins
PI3K-γ: Phosphoinositide 3-kinase
PPAR: Peroxisome proliferator-activated receptor
ROS: Reactive oxygen species
RvD1: Resolvin D1
SR-A: Scavenger receptor-A
SREBP1: Cholesterol regulatory element binding protein 1
SRM: Spermine synthase
STAT: Signal transducer and activator of transcription
TAMs: Tumor-associated macrophages
THP-1: Human monocytic leukemia
TLR: Toll-like receptor
TME: Tumor microenvironment
TNF-α: Tumor necrosis factor-α
